# Isolation and Characterization of the Brassinosteroid Receptor Gene (*GmBRI1*) from *Glycine max*

**DOI:** 10.3390/ijms15033871

**Published:** 2014-03-04

**Authors:** Miao Wang, Shi Sun, Cunxiang Wu, Tianfu Han, Qingyu Wang

**Affiliations:** 1 College of Plant Science, Jilin University, Changchun 130062, Jilin, China; E-Mail: wangmiao2009@yeah.net; 2 Ministry of Agriculture Key Laboratory of Soybean Biology (Beijing), Institute of Crop Sciences, the Chinese Academy of Agricultural Sciences, Beijing 100081, China; E-Mails: sunshi73@163.com (S.S.); wucunxiang@caas.cn (C.W.)

**Keywords:** brassinosteroids, *GmBRI1*, BR receptor, yield, soybean, BR signal transduction

## Abstract

Brassinosteroids (BRs) constitute a group of steroidal phytohormones that contribute to a wide range of plant growth and development functions. The genetic modulation of BR receptor genes, which play major roles in the BR signaling pathway, can create semi-dwarf plants that have great advantages in crop production. In this study, a *brassinosteroid insensitive* gene homologous with *AtBRI1* and other *BRIs* was isolated from *Glycine max* and designated as *GmBRI1*. A bioinformatic analysis revealed that GmBRI1 shares a conserved kinase domain and 25 tandem leucine-rich repeats (LRRs) that are characteristic of a BR receptor for BR reception and reaction and bear a striking similarity in protein tertiary structure to AtBRI1. *GmBRI1* transcripts were more abundant in soybean hypocotyls and could be upregulated in response to exogenous BR treatment. The transformation of *GmBRI1* into the *Arabidopsis* dwarf mutant *bri1-5* restored the phenotype, especially regarding pod size and plant height. Additionally, this complementation is a consequence of a restored BR signaling pathway demonstrated in the light/dark analysis, root inhibition assay and BR-response gene expression. Therefore, *GmBRI1* functions as a BR receptor to alter BR-mediated signaling and is valuable for improving plant architecture and enhancing the yield of soybean.

## 1. Introduction

The soaring demand for more plant-derived products makes a new green revolution essential. Because great success has been achieved by semi-dwarf wheat and rice in the green revolution of the 1960s, enhancing yields and reducing lodging remain the eternal theme [1,2]. The findings that the modification of the gibberellic acid pathway confers such semi-dwarfness highlight the important role of phytohormones in plant development and architecture that underlie high yield [3,4]. Among the phytohormones, the great potential of brassinosteroids (BRs) in agriculture has been recognized, as there is substantial evidence that the genetic modulation of BR signaling can create a semi-dwarf phenotype that provides lodging resistance and, in turn, impacts yield [5,6].

Brassinosteroids (BRs) are a group of steroidal phytohormones with essential roles in plant growth and development, including cell elongation, cell division, skotomorphogenesis, photomorphogenesis, xylem differentiation, seed germination [7] and stress responses [8–11]. In the BR signaling pathway, BRs are perceived by a plasma membrane receptor, *brassinosteroid insensitive 1* (*BRI1*), a leucine-rich repeat (LRR) receptor-like kinase (RLK) [12]. The structure of the BRI1 protein is highly adapted to its function in BR perception and receptor activation. This protein possesses three major domains: a large extracellular domain, a small transmembrane domain and an intracellular kinase domain [13–15]. The extracellular domain contains an amino *N*-terminal signal peptide and a leucine-zipper motif that are important for targeting BRI1 to the plasma membrane and possibly for dimerization. Additionally, the domain with 25 tandem LRRs, which is interrupted by a 70-amino acid island (ID) located between the 21st and 22nd LRRs, and the ID together with the 22nd LRR forms a 94-amino-acid-long steroid binding site for binding BR molecules [16–18]. The intracellular domain can be further divided into a small intracellular juxtamembrane region (JM), a kinase catalytic domain and a *C*-terminal tail. The JM domain is required for transducing the signal from the outside to the inside of a cell. The catalytic domain plays an essential role in the basal activity of BRI1. The *C*-terminal tail apparently plays a negative role in keeping BRI1 in the basal state before BR stimulus [19]. Therefore, in the presence of BRs, BRI1 can perceive BRs via the extracellular domain, after which a series of biochemical events occur, including the autophosphorylation of BRI1 in its *C*-terminal domain, the dissociation of an inhibitory protein, BRI1 kinase inhibitor 1 (BKI1), and the association of BRI1 with another LRR-RLK, BRI1-associated receptor kinase 1 (BAK1) [20,21]. Consequently, a completely active BR receptor complex is formed, inactivating brassinosteroid-insensitive 2 (BIN2) and subsequently activating BRI1-supressor 1 (BSU1), resulting in the activation and nuclear accumulation of brassinazole-resistant 1 (BZR1) and BRI1-EMS-supressor 1 (BES1). The activated BZR1 and BES1 directly bind the promoters of BR-regulated genes to affect their expression [15,22,23].

Recent studies suggest that the genetic modulation of BR receptor genes can alter plant architecture with the potential to impact yield. For example, the rice (*Oryza sativa*) dwarf mutant, *d61-7*, a weak mutant of *OsBRI1*, has a 35% increase in biomass at a high planting density [5]. Moreover, when the endogenous *OsBRI1* expression was partially suppressed, the grain yield of this transgenic line was up to 35% larger than that of the wild-type in close planting, as predicted [5]. As for barley, the *uzu* gene is currently being introduced into all of the hull-less cultivars in Japan, because its semi-dwarf phenotype leads to lodging resistance, which is very suitable for high yields. This *uzu* phenotype is caused by a single nucleotide substitution in *HvBRI1* [6]. Additionally, the semi-dwarf mutant of pea (*lka*) [24,25] and tomato [*cu-3* (*curl-3*)] [26] are also defective in *BRI1* homologs, highlighting the conservation of the function of *BRI1* in BR perception in higher plants. All of the above information suggests the feasibility of generating semi-dwarf plants without defects in reproductive development by modifying the expression of the BR receptor gene.

As an important industrial crop providing oil and protein worldwide, soybean requires the introduction of semi-dwarf cultivars to reduce stature and lodging, as occurs in the cereal crops, rice and wheat. Lodging reduces the soybean harvestable yield by 56% on average and also reduces the grain quality; these situations will worsen when lodging occurs at the anthesis and podding stages [27]. Therefore, yield potential improvement, the principal target for soybean breeders, must include resistance to lodging. In soybean, for high yield breeding, a moderate height and resistance to lodging are the preferred parental characteristics with the potential for high output in close planting [28]. The evolution of crop variety to a large extent is the improvement of plant type [29]. Conventional breeding was the common means of improving plant type in the past, but molecular techniques currently provide a broader space for creation, as a range of genes related to plant height have been identified, including *BRI1*.

Although great progress has been made in the isolation and characterization of the *BRI1* gene involved in the BR signaling pathway, evidence in soybean is limited. In this study, we cloned and characterized a cDNA (*GmBRI1*) corresponding to the *BRI* gene and analyzed the phylogenetic relationships among the BRIs from different plant species. We present sequence, structural and functional evidence that the deduced *GmBRI1* gene has significant homology with *AtBRI1*. Results concerning the expression responses to BR in soybean will increase our understanding of the role of *GmBRI1* in soybean development.

## 2. Results

### 2.1. Isolation of the *BRI1* Gene from Soybean

Initially, we searched the soybean translated nucleotide database of NCBI using the protein sequence of the *Arabidopsis BRI1* gene (*AtBRI1*, GenBank accession No. AAC49810). One gene was identified as homologous with *AtBRI1* and was named *GmBRI1* (Glyma06g15270, GenBank accession No. XM_003526791). The fragment of this putative soybean *AtBRI1* homolog was amplified in *Williams 82* using specific primers (shown in Table S1). Five clones were sequenced, and identical sequences were found. The sequencing results showed that the *GmBRI1* gene contained a 3555-bp open reading frame (ORF) and, like its ortholog in *Arabidopsis*, has no introns.

### 2.2. GmBRI1 Is Strongly Similar in Sequence and Structure to BRIs

**Figure 1.**
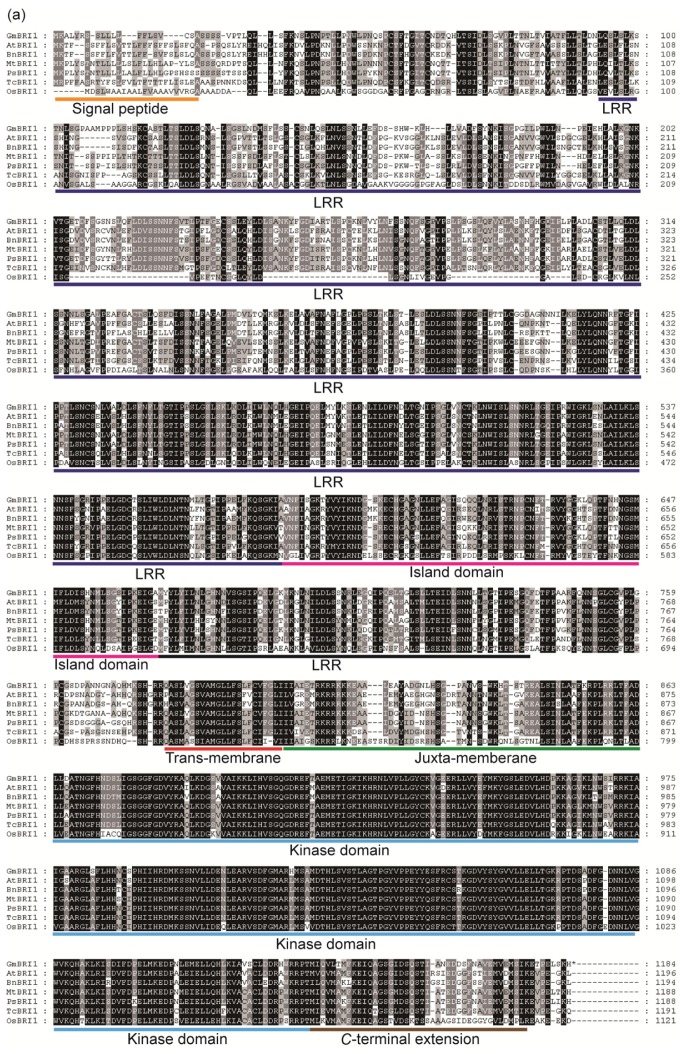

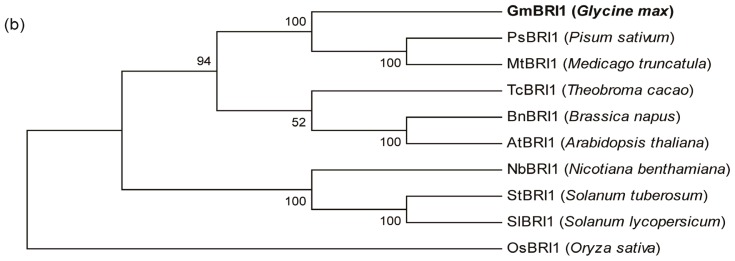
Characterization of GmBRI1 protein. (**a**) Multiple alignment of the deduced GmBRI1 protein with brassinosteroid insensitive (BRIs) from various species (At, *Arabidopsis*; Bn, *Brassica napus*; Mt, *Medicago truncatula*; Ps, *Pisum Sativum*; Tc, *Theobroma cacao*; and Os, *Oryza sativa*). The predicted functional domains are marked in the relative positions. The amino acid sequences were obtained from the NCBI protein database. The amino acid sequences that are identical in all seven molecules are shaded black, while sequences that are conserved in four to six species are shaded gray; and (**b**) Phylogenetic analysis of GmBRI1 and homologs from other organisms. A bootstrap consensus tree was generated using the neighbor-joining method in MEGA 5.02. The bootstrap values from 1000 bootstrap replicates are shown next to the branches. The accession numbers are as follows: GmBRI1, XP_003526839; PsBRI1, BAC99050.1; MtBRI1, XP_003602504.1; TcBRI1, EOX92323.1; BnBRI1, AFU83229.1; AtBRI1, AAC49810; NbBRI1, ABO27628.1; StBRI1, ABO27627.1; SlBRI1, AAN85409.1; OsBRI1, NP_001044077.1.

The *GmBRI1* gene encodes a protein of 1184 residues that has a predicted molecular mass of 129.33 kDa and a pI of 6.27. A multiple alignment revealed that GmBRI1 is highly homologous to BRIs from other plant species (Figure 1a). GmBRI1 was predicted to have 69% identity with AtBRI1, the *Arabidopsis* homolog, 67% identity with BnBRI1, the homolog of another crucifer plant, *Brassica napus*, 80% and 81% identity to MtBRI1 and PsBRI1, the homologs of two legume plants, *Medicago truncatula* and *Pisum sativum*, respectively, 54% identity to OsBRI1, the *Oryza sativa* homolog and 73% identity to TcBRI1, the *Theobroma cacao* homolog. Because the conserved amino acids were present at the expected positions in the deduced GmBRI1 sequence, the identified gene may possess the expected function of a BR receptor.

Additionally, a phylogenetic tree was constructed to investigate the evolutionary relationships among BRIs (Figure 1b). From the data, we can find that GmBRI1 is closely related to the BRIs from cruciferae plants and is much more closely related to the BRIs from legume plants. The BRIs of dicotyledons fall into one group. These results further suggest that BRIs were derived from a common ancestor based on their conserved structure and sequence characteristics.

**Figure 2.**
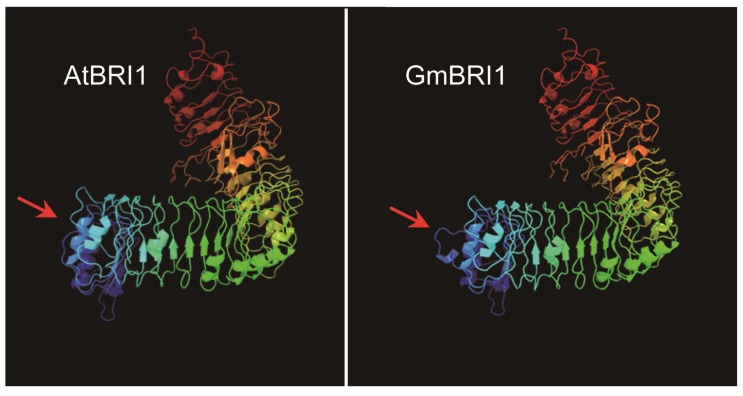
Predicted tertiary protein structure of the GmBRI1 and AtBRI1 proteins. Arrows indicate the differing region.

A structural analysis showed that the GmBRI1 polypeptide includes all of the functional domains contained in AtBRI1 (Figure 1a). The kinase domain of GmBRI1 has 92% identity with that of AtBRI1. Considering that the entire sequence identity between GmBRI1 and AtBRI1 is 69%, the similarity of the kinase domain is extremely strong. Conversely, the signal peptide domain varies among species and is 53% similar between GmBRI1 and AtBRI1. The presence of 25 tandem LRRs, the most characteristic feature of AtBRI1, is also found in GmBRI1 with an identity of 64% (Figure 1a). Furthermore, the tertiary structure of the protein was predicted in order to investigate the structural similarity between GmBRI1 and AtBRI1 (Figure 2). These proteins could be considered one molecular model, except for minor differences that exist in the *N*-terminus (Figure 2, blue part). According to the sequence analysis, the differing region includes a signal peptide domain, which is involved in protein processing and membrane localization. The differences may due to diverse mechanisms among species. At all of the events, the functional structure involved in the reception and in kinase activity is highly conserved. All of the above information indicates that GmBRI1 possesses all of the structural characteristics of a typical leucine-rich repeat receptor-like kinase. Additionally, the high degree of structural similarity indicates that GmBRI1 and AtBRI1 are similar in function.

### 2.3. Expression of *GmBRI1* Gene in the *Arabidopsis* Mutant Complements the Phenotype

**Figure 3.**
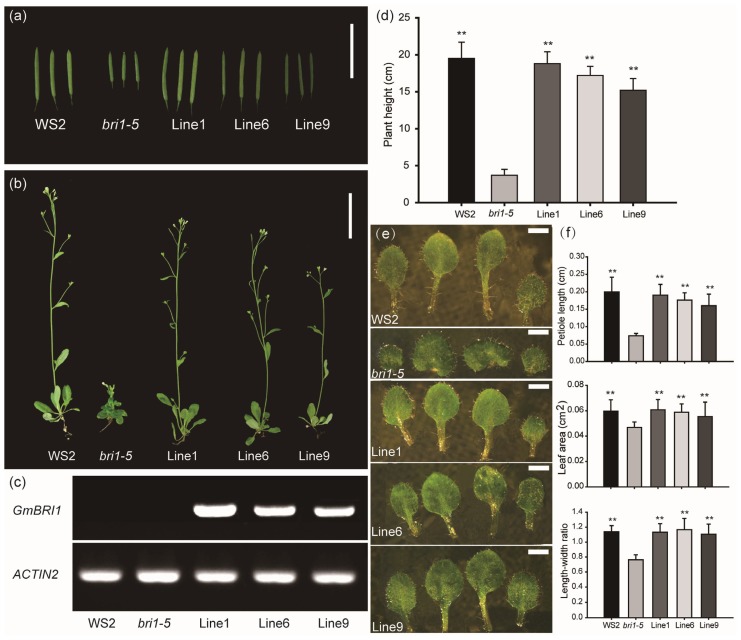
*GmBRI1* complementation of *bri1-5* in the phenotype. (**a**) Silique morphology comparison among the wide-type (Waardenburg syndrome type 2, WS2), *bri1-5* mutant and three transgenic lines (Line 1, 6 and 9). Bar = 10 mm; (**b**) Phenotype comparison of adult plants. Bar = 40 mm; (**c**) Real-time polymerase chain reaction (RT-PCR) analysis to detect the *GmBRI1* gene using the specific primers described in Table S1, *AtACTIN2* (GenBank accession No. AT3G18780) as a reference gene; (**d**) Plant height statistics with a sample capacity of 20 plants per group; (**e**) Leaf phenotypes from 13-day-old plants. Seedlings were photographed under a Nikon SMZ1500 dissection microscope using a Nikon DS-Fi1 camera (Nikon, Mississauga, ON, Canada) and NIS-Elements F Imaging software (Nikon, Mississauga, ON, Canada). Bar = 1 mm; and (**f**) Statistical analysis of leaf measurements: the petiole length, leaf area and length-width ratio of the first true leaves. The data represents the mean ± SD of three independent experiments. The asterisks indicate significant differences compared to the *bri1-5* mutant (**, *p* < 0.01 by the *t*-test).

To confirm that the BR receptor protein encoded by *GmBRI1* is functional, the *GmBRI1* coding sequence under the control of the *CaMV 35S* promoter was transformed into the *Arabidopsis* brassinosteroid-insensitive dwarf mutant, *bri1-5* [30]. The *bri1-5* plants are severely dwarfed with short rounded leaves and very short internodes along the inflorescence (Figure 3b). Comparatively, the transgenic *Arabidopsis* plants of *GmBRI1* are similar in size and morphology to the wild-type Waardenburg syndrome type 2 (WS2) plants (Figure 3). As expected, the polymerase chain reaction (PCR) analysis showed that the *GmBRI1* transgene could only be detected in the transgenic plants, but not in the WS2 wild-type or *bri1-5* mutant plants (Figure 3c).

Unlike the leaf blades seen in the WS2, the *bri1-5* mutant produces rounded curled leaves and shortened petioles (Figure 3e). The morphologies of the leaf blade in transgenic lines are observed to be similar to the WS2 type (Figure 3e). In the quantitative comparison, the *bri1-5* mutant possesses shorter petiole length, smaller leaf area and lower length-width ratio compared with either the wild-type or transgenic lines (Figure 3f). Student’s *t*-tests indicate that the measurements between the transgenic plants and *bri1-5* mutant are significantly different (*p* < 0.01) (Figure 3f).

**Figure 4.**
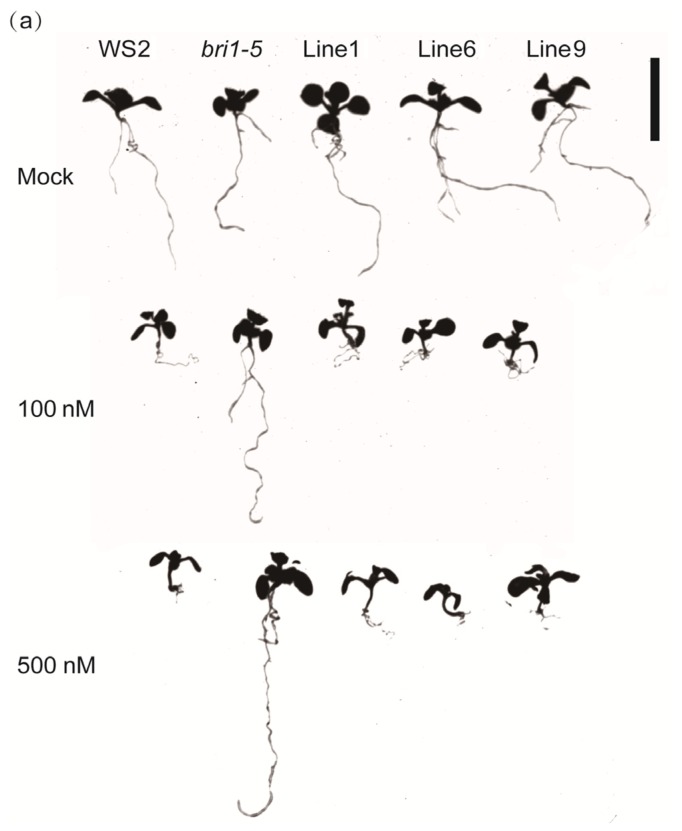

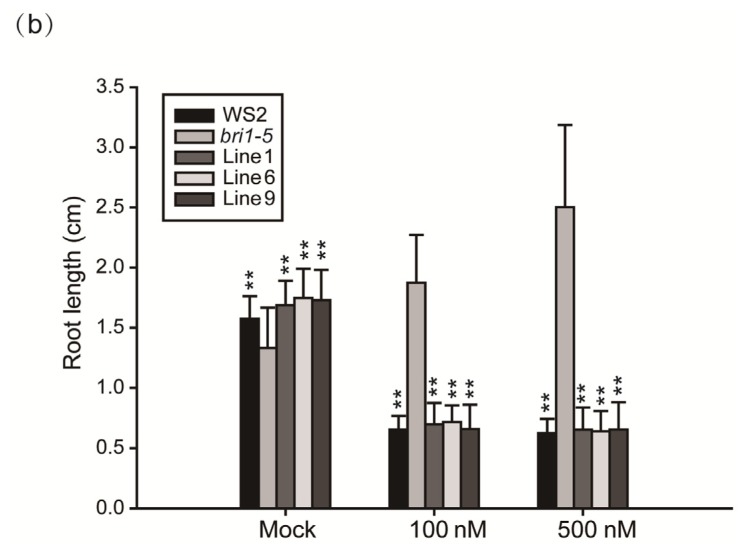
Root inhibition assays showing different brassinosteroid (BR) responses in the wide-type, *bri1-5* mutant and transgenic lines. (**a**) Phenotypes of the wide-type (WS2), *bri1-5* mutant and three transgenic lines (Line 1, 6 and 9) were grown in half-strength MS medium supplemented with 1% sucrose (Mock), 100 and 500 nM 2,4-epibrassinolide (24-epiBL) for 10 days. The images were scanned by an Epson perfection V700 photo scanner (Epson, Nagano, Japan) and WinRHIZO Pro v.2009c software (Regent Instruments, Montreal, QC, Canada). Bar = 10 mm; and (**b**) Measurements of the total root length of the seedlings shown in (**a**). The data represents the mean ± SD of three independent experiments. The asterisks indicate significant differences compared to the *bri1-5* mutant (**, *p* < 0.01 by the *t*-test).

In the root analysis, total root length of the 10-day-old plants was compared between the WS2 plant, *bri1-5* mutant and transgenic lines. As shown in the mock of Figure 4, the root length of the transgenic lines is similar to the WS2 plants and is longer compared to *bri1-5* mutant. Student’s *t*-tests indicate a significant difference (*p* < 0.01) between the transgenic lines and the mutant plants (Figure 4b Mock).

Particularly, the silique size is decreased in *bri1-5*, while the transgenic lines are similar to the wild-type, with long and strong siliques (Figure 3a). Dwarfism, the characteristic phenotype of BR-related mutants, such as *bri1-5*, is restored by the transformation of *GmBRI1* (Figure 3b,d).

All these results indicate that the *GmBRI1* transgene successfully complements the BR-insensitive phenotype.

### 2.4. Expression of *GmBRI1* Gene in the *bri1-5* Restores the BR Signaling Pathway

To further confirm that the phenotypes of the transgenic plants are a consequence of a restored BR signaling pathway, we examined the BR signaling output of the transgenic plants compared with non-transformed *bri1-5* and WS2 wild-type.

The balanced BR signaling is needed for the optimal root growth [31]; root inhibition assays were carried out to examine whether the inhibited BR signaling output in the *bri1-5* mutant was rescued by *GmBRI1* gene transformation. In the current assays, seedlings of the transgenic lines, *bri1-5* and WS2 were planted in half-strength MS medium supplemented with 1% sucrose as the control (Mock), and they were also planted in half-strength MS medium with 1% sucrose and two concentrations, 100 and 500 nM, of 2,4-epibrassinolide (24-epiBL). Then the total root length was measured after 10 days. According to the previous studies, exogenous application of BRs appears to promote root growth at low concentrations, but inhibit it when higher than 0.04 nM [31–34]. In our results, the root development of the WS2 plants responding to 24-epiBL is in agreement with the above observations: the root length of WS2 is much smaller in either 100 or 500 nM 24-epiBL compared with the Mock (Figure 4). By contrast, the high concentration of 24-epiBL promotes root growth in the *bri1-5* background (Figure 4). The total root length of the *bri1-5* are increased by about 41% in 100 nM and increased by about 34% in 500 nM 24-epiBL than the Mock (Figure 4b). As shown, the transgenic lines exhibit a similar 24-epiBL response to the wild-type, which show shorter root length under the high concentration of 24-epiBL and have significant differences (*p* < 0.01) compared with the *bri1-5* mutant (Figure 4). These results are consistent with the hypothesis that the BR signaling is restored by the expression of the *GmBRI1* gene in the *bri1-5* mutant background.

**Figure 5.**
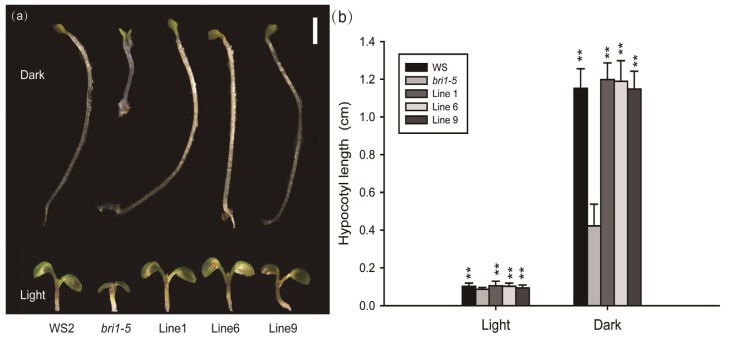
*GmBRI1* restores hypocotyl elongation of *bri1-5*. (**a**) Morphologies of the six-day-old seedlings grown in light and darkness. Representative seedlings are the wild-type (WS2), *bri1-5* mutant and three transgenic lines (Line 1, 6 and 9) from left to right. Seedlings were photographed under a Nikon SMZ1500 dissection microscope using a Nikon DS-Fi1 camera and NIS-Elements F Imaging software. Bar = 1 mm; and (**b**) Average hypocotyl length of seedlings grown for six days in light and darkness. The data represents the mean ± SD of three independent experiments. The asterisks indicate significant differences compared to the *bri1-5* mutant (**, *p* < 0.01 by the *t*-test).

Then, we screened for the transgenic lines, *bri1-5* mutant and wild-type in hypocotyl elongation during skotomorphogenesis and photomorphogenesis. Seedlings were germinated and grown for six days in the dark and light separately in half-strength Murashige and Skoog (MS) medium supplemented with 1% sucrose, then the hypocotyl length were measured. In the dark, the *bri1-5* mutant exhibits short hypocotyl and open cotyledons, whereas the hypocotyl is elongated, and the hook of cotyledons is closed in the wild-type and transgenic lines (Figure 5a). In the light, the *bri1-5* mutant shows significantly shorter hypocotyl compared with the wild-type and transgenic lines (Figure 5a). However, the transgenic lines are phenotypically indistinguishable from the wild-type in both dark and light. Student’s *t*-tests indicate a significant difference (*p* < 0.01) in hypocotyl length between the transgenic lines and mutant plants (Figure 5b). Therefore, *GmBRI1* was found to have restored hypocotyl elongation in the *bri1-5* mutant, which is positively regulated by BRs [35,36].

**Figure 6.**
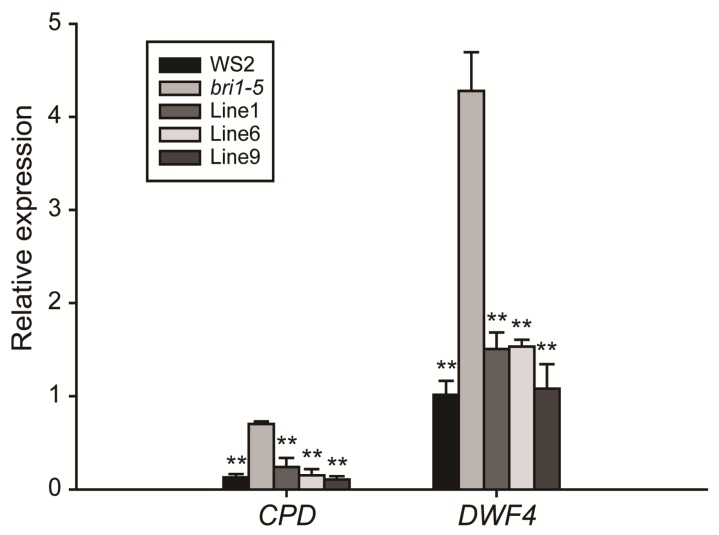
Real-time quantitative reverse transcriptase polymerase chain reaction (qRT-PCR) analysis of the BR-related marker genes, *DWF4* and *CPD*, in the wide-type (WS2), *bri1-5* mutant and three transgenic lines (Line 1, 6 and 9). The relative expression levels are normalized to *AtACTIN2* (GenBank accession No. AT3G18780). The data represents the mean ± SD of three independent experiments. The asterisks indicate significant differences compared to the *bri1-5* mutant (**, *p* < 0.01 by the *t*-test).

Furthermore, we measured the expression levels of the BR-related marker genes, *DWF4* and *CPD*. The transcript levels of these two BR biosynthetic genes should be downregulated by feedback inhibition in response to BR signaling [37,38]. As shown in our examination of the 13-day-old wild-type, *bri1-5* mutant and transgenic lines, the transcript levels of *DWF4* and *CPD* are significantly (*p* < 0.01) downregulated in the *bri1-5* with the transformed *GmBRI1* gene compared with the non-transformed one (Figure 6). Therefore, the results suggest that the rescued phenotype of the *bri1-5* mutant may be attributed to the restored BR signaling pathway by the functional *GmBRI1* gene.

### 2.5. Expression of *GmBRI1* in Different Soybean Organs

**Figure 7.**
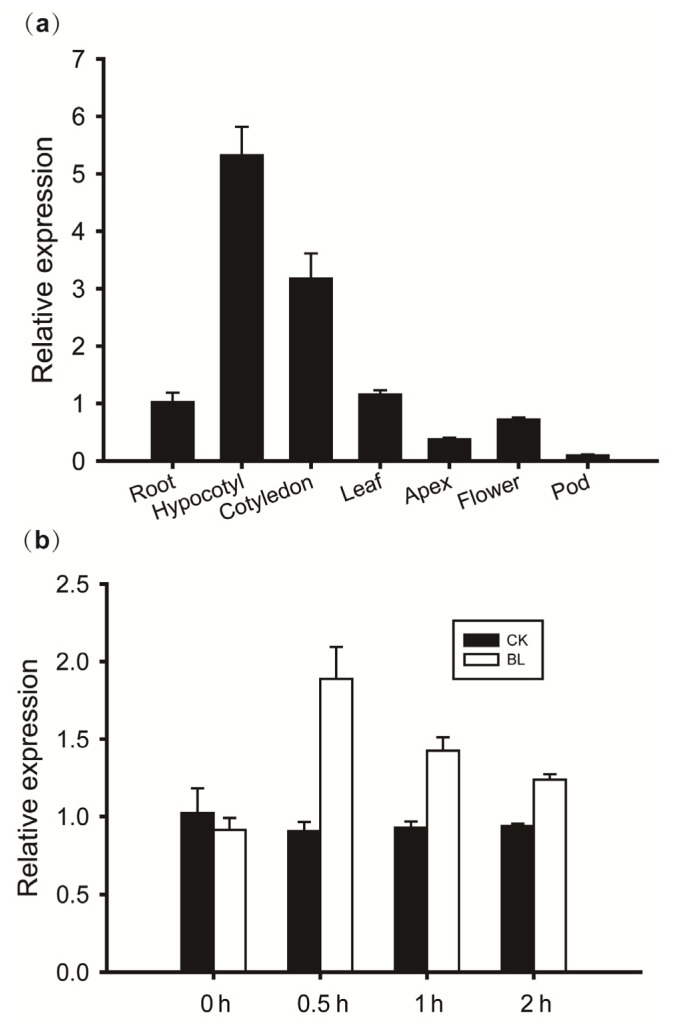
(**a**) qRT-PCR analysis of *GmBRI1* mRNA in different tissues of soybean cv. *Williams 82*. The sampling time of each tissue is described in Section 4.3. The relative expression levels are normalized to *GmELF* (GenBank accession No. NM_001249608). The data represent the mean ± SE of three independent experiments; and (**b**) The inducible expression of *GmBRI1* in the leaves of *Williams 82* under BR treatment. CK, untreated group (black column); BR, treated group (white column). The *x*-axis represents the time of the treatment: 0 h (before treatment), 0.5 h (treated for 0.5 h), 1 h (treated for 1 h) and 2 h (treated for 2 h). The relative expression levels are normalized to *GmG6PDH* (GenBank accession No. XM_003547631). The data represent the mean ± SE of three independent experiments.

Quantitative real-time PCR was performed to survey the gene expression pattern of *GmBRI1* throughout the soybean plant (Figure 7a). For this analysis, total RNA samples were isolated from different parts of the soybean plants, including the root, hypocotyl, cotyledon, leaf, shoot apex, flower and pod. *GmBRI1* displays extremely high mRNA levels in the hypocotyl and cotyledon. Its expression also can be detected in other organs, but at a much lower level, with the pod exhibiting the lowest level. Therefore, because the hypocotyl and cotyledon as rapidly growing tissues undergo cell expansion and/or vascular development, a high level of *GmBRI1* expression is expected.

### 2.6. Transcript Levels of *GmBRI1* in Response to Brassinosteroids (BRs) in Soybean Plants

To confirm the role of *GmBRI1* in sensing the BR signal, its transcript levels in soybean leaves were monitored via qRT-PCR over a two-hour period following 24-epiBL application. Samples were collected from both the treated and untreated (control) groups every hour, including 0 h (before treatment), 0.5 h (treated for 0.5 h), 1 h (treated for 1 h) and 2 h (treated for 2 h) for each group. As demonstrated by the results, *GmBRI1* is clearly responsive to 24-epiBL, with fluctuating mRNA levels. At the beginning of the treatment (0.5 h), the mRNA level peaks dramatically and then smoothly decreases (Figure 7b). However, in the long run, the *GmBRI1* expression levels are upregulated during two-hour treatment comparing to that of the control. From these results, according to the 0.5 h peak, we can imply that *GmBRI1* is very sensitive to exogenous BR, supporting its receptor role in BR signaling.

## 3. Discussion

Since brassinosteroids (BRs) were first isolated and purified from *Brassica napus* pollen in 1979 [39] and widely recognized as a novel phytohormone in the 1990s, components of BR signaling and biosynthesis have been identified in a broad range of species, but rarely in soybean, an economically important legume crop. In the 1990s, only two soybean genes, *BRU1* [40,41] and *SMT* [42], were identified. This study is the first time that the *BRI1* gene was isolated and characterized in soybean. Because the *BRI* gene plays a major role in BR signaling, which participates in plant development, and because the relationship between *BRI* and plant yield needs to be understood to further benefit production, the isolation and characterization of *GmBRI1* is only the beginning.

The complementation of the *bri1-5* mutant highlights the functionality of the *GmBRI1* gene that we isolated from soybean. *GmBRI1* expression in *bri1-5* causes changes in the pod size and plant height, indicating that these two important traits could be controlled through *GmBRI1* gene modulation. There may exist a balance in which the plant height is reduced to a level to satisfy the production demands while maintaining or even increasing the yield. The evidence in rice that modifying the expression of the *OsBRI1* gene can be used to generate semi-dwarf plants without defects in reproductive development supports this hypothesis [5]. Consequently, our data provide a foundation for future studies into the regulation of soybean production by BR signaling. The successful deciphering of the *GmBRI1* genes will contribute toward generating new soybean germplasm.

## 4. Experimental Section

### 4.1. Plant Material and Growth Conditions

As a genome-sequenced soybean (*Glycine max*) variety, *Williams 82* was chosen. The plants were grown in a chamber under the following conditions: 12/12 h day/night cycle, 26/24 °C day/night temperature, 60% humidity and 250 μmol·m^−2^·s^−1^ light.

*Arabidopsis* plants (ecotype WS2 and *bri1-5*) were grown in potting soil under the following conditions: 16/8 h day/night cycle, 22 °C temperature, 50% relative humidity (RH) and 250 μmol·m^−2^·s^−1^ light.

For some assays, *Arabidopsis* plants were axenically cultured and grown vertically in half-strength MS medium supplemented with 1% sucrose under the conditions as above in light or darkness (packed by silver papers).

### 4.2. Brassinosteroid Treatment

The 5-day-old light-grown soybean seedlings were germinated in soil and then cultivated in Hoagland solution. Soybean plants (15 days after germination) were treated with 1 μM 2,4-epibrassinolide (C28H48O6; TCR, Toronto, ON, Canada) for 2 h, and the samples were collected every hour with a 0.5 h point added. As a control, corresponding mock treatments were carried out concomitantly. The treated samples were frozen immediately in liquid nitrogen and stored at −80 °C until the RNA extraction. Each treatment was performed in replicate.

In the root inhibition assay, *Arabidopsis* seeds were planted on vertically oriented plates containing half-strength MS medium supplemented with 1% sucrose in the absence or presence of 100 or 500 nM 24-epiBL. Seedlings were grown for 10 days, then the root length was measured.

### 4.3. DNA and RNA Isolation

Soybean and *Arabidopsis* genomic DNA samples were isolated from the leaf tissues using the DNeasy Plant Mini Kit (Qiagen, Hilden, Germany). Soybean total RNA samples were isolated from the leaves, stems and shoot apices of soybean plants (20 days after germination) and the hypocotyls, cotyledons and roots of seedlings (7 days after germination). The flowers were tagged on the day of anthesis, and the pods were harvested when 0.5–2 cm long. The samples were ground into powder in liquid nitrogen, and the total RNA was extracted using TRIzol Reagent (Invitrogen, Carlsbad, CA, USA). The *Arabidopsis* RNA was isolated from entire plant tissues using the same methods.

### 4.4. Sequence Analyses

The protein sequences of *GmBRI1* were deduced from the cDNA, and the NCBI database was searched for homologous proteins using protein BLAST. The sequences with high homology to GmBRI1 were aligned using Clustal W2. The neighbor-joining (NJ) method of phylogenetic tree reconstruction was performed using MEGA 5.02. The tertiary protein structure was predicted by PHYRE (http://www.sbg.bio.ic.ac.uk/~phyre).

### 4.5. Measurements and Statistical Analysis

All the seedlings axenically cultured on medium for the light/dark analysis, root inhibition assay and leaf morphology analysis were scanned using an Epson perfection V700 photo scanner (Epson, Nagano, Japan), and the images were analyzed by WinRHIZO Pro v. 2009c software (Regent Instruments, Montreal, QC, Canada). For the light/dark analysis, the 6-day-old seedlings were used, and the hypocotyl length was measured. Similarly, the total root length of 10-day-old seedlings grown on the medium with or without 24-epiBL was measured in the root inhibition assay. For the leaf morphology analysis, the first true leaves of 13-day-old seedlings were cut off at the bottom of the petioles and flattened on agar plates to scan. The traits, including the petiole length, leaf area, length and width of the leaf blade, were examined. All measurements were repeated three times independently, and 30–50 seedlings were measured each time. For comparisons, Student’s *t*-tests were performed in all cases.

### 4.6. *GmBRI1* Transgene Complementation

The cloned *GmBRI1* gene was inserted into pTF101.1-GFP in place of the GFP gene. *Xba*I and *Kpn*I were chosen as the restriction enzyme cutting sites. The ligated plasmids were transformed into *Escherichia coli*. The pTF101.1-GmBRI1 plasmids were identified by sequencing and restriction analysis and were transformed into *Agrobacterium tumefaciens* GV3101. *A. tumefaciens* containing the pTF101.1-GmBRI1 construct was selected on lysogeny broth (LB) plates containing kanamycin (50 mg/L), spectinomycin (50 mg/L), chloramphenicol (25 mg/L) and rifampicin (20 mg/L). The plasmid was isolated, and the insert was sequenced to confirm the accuracy of the *GmBRI1* transgene.

The *GmBRI1* transgene was introduced into the *bri1-5* mutant *Arabidopsis* plants by the *Agrobacterium*-mediated flower infiltration transformation method [43]. The seeds were harvested from individual plants. Next, the *Arabidopsis* seeds were sterilized with 50% chlorine bleach solution for 15 min. The seeds were then washed with sterile water 3–5 times and sown on antibiotic-containing MS plates with 250 mg/L carbenicillin, 250 mg/L cefotaxime and 10 mg/L glufosinate ammonium (Sigma, St. Louis, MO, USA). A PCR analysis with primers specific for the *CaMV 35S* promoter and the *GmBRI1* coding sequence was performed to confirm the insertion of the *GmBRI1* transgene. T_3_ transgenic plants were obtained for investigation.

### 4.7. Analysis of the *GmBRI1* Gene Expression

A qRT-PCR was performed on ABI7900 (Applied Biosystems, Foster City, CA, USA) using the Takara SYBR Premix ExTaq (Takara, Shiga, Japan). Each reaction was run in a 10-μL volume with 2 μL of cDNA. All of the PCR reactions were performed under the following standard conditions: 95 °C for 10 s, followed by 40 cycles of 95 °C for 5 s and 60 °C for 30 s. The results were analyzed using the SDS 2.3 software (Applied Biosystems, Foster City, CA, USA). Relatively, the RT-PCR was performed on an Eppendorf Mastercycler (Eppendorf, Hamburg, Germany) using the Promega PCR Master Mix (Promega, Madison, WI, USA). Each reaction was run in a 20-μL volume with 2 μL cDNA. The PCR cycling conditions consisted of an initial denaturation at 94 °C for 5 min, then 35 cycles of denaturation at 94 °C for 30 s, annealing at 55 °C for 30 s and elongation at 72 °C for 30 s, followed by a final elongation step at 72 °C for 10 min. The specific primers for each gene are shown in Table S1.

## 5. Conclusions

The deduced GmBRI1 has 69% identity with *Arabidopsis* AtBRI1. A sequence analysis indicated that the GmBRI1 protein contains a kinase domain and 25 tandem LRRs, which are conserved among plant BRIs and are important for BR binding and reaction. The GmBRI1 protein is strikingly similar to AtBRI1 in the prediction of its tertiary structure. A phylogenetic analysis suggested that GmBRI1 is closest in homology to BRIs from dicotyledons, especially leguminous plants. Additionally, the transformation of *GmBRI1* into the *Arabidopsis bri1-5* mutant, restored the phenotype, especially regarding pod size and plant height. Additionally, this complementation is a consequence of a restored BR signaling pathway. These results support *GmBRI1* as a novel *BRI1* gene. Furthermore, the high level of *GmBRI1* transcript in soybean rapidly growing tissues, such as hypocotyls, and its response to exogenous BR treatment suggest the vital role of *GmBRI1* in soybean development. Further investigations are needed to confirm the relationship between *GmBRI1* and plant architecture, which could lead to the potential development of soybean yield.

## Supplementary Information
