# Supplementary material for: Isolation and Characterization of the Brassinosteroid Receptor Gene (GmBRI1) from Glycine max
Source: Int J Mol Sci. 2014 Mar 4;15(3):3871–88. doi: 10.3390/ijms15033871 (PMC3975373; doi:10.3390/ijms15033871)
Supplement: Supplementary file 1 [file ijms-15-03871-s001.pdf]

Supplementary Information

Table S1. Primer sequences used.

Role	Primer name	5' to 3' sequence
gene cloning	GmBRI1-S	CTTAACCTTTCACCTTCCATATCTG
gene cloning	GmBRI1-A	TGTCTGTTTCCCAAAGAATCCAC
qRT-PCR	qGmBRI1-S	TCTCCGTCTGTTCTGCATCTTCTT
qRT-PCR	qGmBRI1-A	GAGGTCTATGGAAGTGAGGTGCTG
qRT-PCR	GmG6PDH-S	GTCTGTTATCCGCCTACAGCCT
qRT-PCR	GmG6PDH-A	ACTCCTTGATACCGTTGTCCAT
qRT-PCR	GmELF1B-S	GTTGAAAAGCCAGGGGACA
qRT-PCR	GmELF1B-A	TCTTACCCCTTGAGCGTGG
qRT-PCR	AtDWF4-S	TTCTCGTTATGACCAACCTAATCTC
qRT-PCR	AtDWF4-A	AGGATGACGCTCCGTTGTT
qRT-PCR	AtCPD-S	CCTTGGAGATGGCAGCAA
qRT-PCR	AtCPD-A	GTAACCGGGACATAGCCTTG
qRT-PCR, RT-PCR	AtACTIN2-S	ACTCTCCCGCTATGTATGTCGC
qRT-PCR, RT-PCR	AtACTIN2-A	AGAAACCCTCGTAGATTGGCAC

© 2014 by the authors; licensee MDPI, Basel, Switzerland. This article is an open access article distributed under the terms and conditions of the Creative Commons Attribution license (http://creativecommons.org/licenses/by/3.0/).
